# Scotland

**Published:** 1907-09-07

**Authors:** 


### SCOTLAND.

*The Carnegie Trust and the Payment of Class Fees*.—This is a fund to provide under certain regulations necessitous but deserving students with all or a portion of the class fees of the curriculum.

Applicants must be over 16 years of age, and of Scottish birth or extraction.

*University of Edinburgh*.—Clinical instruction is given in the Royal Infirmary, which contains nearly 900 beds; Royal Hospital for Sick Children, with 120 beds; Maternity Hospital; City Fever Hospital; and the Royal Morningside Asylum.

A Diploma in Tropical Medicine and Hygiene has recently been instituted. It is conferred on graduates of the University after an approved course of study and examinations in Tropical diseases, bacteriology, medical entomology and protozoology, and clinical Tropical medicine.

*School of Medicine of the Royal Colleges, Edinburgh*.—This school of medicine is constituted by an association of lecturers who lecture in several buildings near the Royal Infirmary. The lecturers are recognised or licensed by the Royal Colleges of Surgeons and Physicians, and also by the University.

The teaching is similar to that of the Scottish Universities, and the students receive similar certificates at the close of each session. The lectures qualify for the University of Edinburgh and other Universities; the Royal Colleges of Physicians and Surgeons of Edinburgh, London, and Dublin; the Faculty of Physicians and Surgeons of Glasgow, and the other Medical Boards. The whole education required for graduation at the University of London may be taken in this school. The Anatomy Rooms and Laboratories open on October 1 and the lectures begin on October 15.

The facilities for clinical instruction are similar to those provided for the University, and there are special classes for women. Full particulars and copy of the calendar of the school may be had gratis from the Secretary, R. N. Ramsay, solicitor, 27 Forrest Road, Edinburgh.

*Medical College for Women, Edinburgh*.—Precisely the same facilities for medical study are given as to male students in the School of Medicine, Edinburgh. Regulations, fees, and arrangements are the same. The lecturers are recognised by the University of Edinburgh as giving courses admitting to the degrees of M.B., Ch.B. Clinical instruction is given in the Royal Infirmary and other hospitals mentioned above.

The benefit of the Carnegie Trust is open to students of the college.

*University of Glasgow.*—The whole course of study for the M.B., Ch.B., can be passed in the Medical School of the University.

The regulations are similar to those given above for Edinburgh. Clinical instruction is given at the Western Infirmary, which contains 416 beds, and also at the Lunatic Asylum, the Eye Infirmary, the Maternity Hospital, the City Fever Hospitals, and various other hospitals.

The cost of the course, including matriculation, fees for classes, for hospital attendance, and for professional examinations, amounts to about £150.

*Queen Margaret College, Glasgow (the Women's Department of the University).*—This is an integral part of the University of Glasgow. The courses, regulations, and fees are the same as for men. The instruction is given by University Professors and Lecturers appointed by the University Court. The College provides a separate building, with class-rooms, laboratories, library, and other teaching appliances. The women have all the rights and privileges of University students, and do their clinical work in the Royal Infirmary, where special wards are reserved for their use, and in other hospitals.

*Anderson's College Medical School, Glasgow.*—The college, at which a full medical curriculum can be obtained, is situated in Dumbarton Road, close to the Western Infirmary and within four minutes' walk of the University.

Degrees and diplomas: Certificates of attendance on the lectures are accepted by the Universities of London and Durham, by the Royal University of Ireland, by the Universities of Glasgow, Edinburgh, etc., under conditions stated in the calendars, and by all the Royal Colleges and licensing boards in the United Kingdom. The Public Health course qualifies for the Scottish Licensing Board, the Irish Colleges, and the University of Cambridge.

The Carnegie Trust extends its benefactions to students of Anderson's College. Particulars may be obtained from W. S. McCormick, Esq., LL.D., The Carnegie Trust Offices, Edinburgh.

A Calendar will be sent on receipt of a postcard by the *Secretary to the Medical Faculty, Anderson's College Medical School, Glasgow, W.*

*St. Mungo's College: the Medical School of the Glasgow Royal Infirmary.*—The College buildings are situated within the grounds of the Infirmary, and are provided with class-room and laboratory accommodation for several hundred students. Students receive their clinical instruction in the wards of the infirmary, which contains over 600 beds. The College provides a complete medical curriculum, and the classes qualify for the diplomas of the English, Scottish, and Irish Conjoint Boards, and under certain conditions for the various universities. Students who have fulfilled the conditions of the Carnegie Trust are eligible for the benefits of this Trust during the whole course of their studies at St. Mungo's College. The classes at St. Mungo's College are open to male and female students equally. Students entering for the dental diploma can take out almost all their classes at St. Mungo's College. There is a special department of public health, attendance on which qualifies for the conjoint boards and the Universities of Oxford and Cambridge. The inclusive fees for the whole medical curriculum amount to about £65.

